# Supplementary material for: Are carbon emissions trading and green financial instruments synergistic? -Comprehensive quantitative research based on content analysis
Source: PLoS One. 2024 Mar 7;19(3):e0298601. doi: 10.1371/journal.pone.0298601 (PMC10919601; doi:10.1371/journal.pone.0298601)
Supplement: S1 Data — (ZIP) [file pone.0298601.s001.zip › Data/地级市Z_F工作报告.zip!/║■▒▒/╥╦▓2╩╨/└┤╫╘╣1⁄2╓┌║┼í╛┬φ┐╦╩2╛▌═°í┐_2012.docx]

各位代表：

　　现在，我代表市人民政府，向大会报告2011年和过去五年工作，提出今后五年目标任务以及2012年工作重点，请予审议，并请市政协委员和列席人员提出意见。

　　一、2011年和过去五年工作回顾

　　2011年，在市委正确领导下，在市人大和市政协监督支持下，我们紧紧依靠全市人民，认真践行科学发展观，团结奋斗，务实拼搏，各项工作都取得新的成绩。实现生产总值2140.7亿元，同比增长16.1%。一般预算收入114亿元，增长37.1%；全社会固定资产投资1190.5亿元，增长33.1%；社会消费品零售总额636亿元，增长18.3%；城镇居民人均可支配收入16451元，增长15.2%；农民人均纯收入7055元，增长18%。实现了“十二五”时期良好开局。

　　刚刚过去的五年，是宜昌发展史上极不平凡的五年。五年来，市政府团结全市人民，沉着应对国内外发展环境的复杂变化和各种重大风险挑战，承前启后，开拓创新，克难奋进，砥砺前行，全市经济、政治、文化、社会和生态文明建设取得重大进展，掀开了宜昌科学发展、跨越式发展新的一页。

　　这五年，是宜昌综合实力快速攀升的五年。坚持把发展作为第一要务，着力构建现代产业体系，三次产业协调发展，经济总量居中部地区和长江沿线同等城市前列，区域性经济中心地位开始显现。2011年，全市生产总值、一般预算收入分别比2006年增长2.1倍和3.1倍。注重区域统筹，城乡经济协调发展。城区经济日益壮大，总量超过全市的51%，辐射带动作用更趋明显。县域经济发展迅猛。宜都、夷陵、枝江、当阳稳居全省县域经济先进行列。宜都进入中部地区县域经济20强。2011年，宜都、夷陵、枝江、当阳生产总值分别突破200亿元，财政总收入均超过15亿元。

　　工业经济蓬勃发展。强力推进工业强市，工业结构不断优化，优势产业快速崛起，工业经济成为市域经济脊梁。2011年，规模以上工业企业达到865家，增加值突破1100亿元、五年年均增长24.6%。化工、食品医药产业快速壮大，装备制造业突飞猛进，电力产业稳中求进，四大支柱产业产值占全市工业比重超过80%。建材、纺织等传统产业升级发展。生物、新材料、新能源、电子信息、节能环保等新兴产业茁壮成长。高新技术产业发展加快，2011年实现增加值204亿元，比2006年增长4.3倍。国家高新技术企业达到74家。自主创新能力增强，建立国家级企业技术中心4家。优强企业倍增工程扎实推进，宜化、兴发、东阳光等一批骨干企业做大做强，年主营业务收入过100亿元的企业达到6家，过10亿元33家。新增中国驰名商标15件，达到19件。新增专利授权3894件。

　　农村经济稳步发展。切实把“三农”工作摆在重中之重，加大农业结构调整力度，现代特色农业产业体系初步形成。粮食连续八年增产。水果、茶叶、蔬菜、畜牧、水产、中药材六大特色产业占农业总产值的75%以上，成为全国重要的柑橘、茶叶、高山蔬菜生产基地。2011年实现农业增加值240.8亿元，比2006年增长1.7倍。农业产业化向纵深推进，规模以上农产品加工企业达到295家，农民专业合作社1673家，农产品加工年产值750亿元以上。夷陵、宜都、枝江农产品加工园成为省级重点园区。稻花香集团成为全省首家年销售过百亿的农业产业化龙头企业。农村水利基础设施建设加强，完成病险水库除险加固70座，新建、改造末级渠系3908公里，新增节水灌溉面积26.8万亩。农业机械化作业综合水平达到57.2%。

　　第三产业全面提速。着力推动旅游业转型升级，旅游服务功能逐步完善。三峡大坝、三峡人家景区升级为5A景区。清江画廊、柴埠溪等景区建设步伐加快。夷陵、长阳、五峰、秭归进入“湖北旅游强县”行列。三斗坪、车溪等一批旅游名镇名村相继涌现。推进三峡区域旅游合作，和重庆共同举办了两届中国长江三峡国际旅游节。2011年，接待国内外游客1930万人次，旅游收入148亿元，分别比2006年增长1.1倍和1.2倍。三峡现代物流中心建设纳入国家三峡后续工作规划，进入全面实施阶段。港口、铁路物流园区建设进展顺利。万达广场、金东山市场等一批商业项目建成营运。“万村千乡市场工程”、“新农村现代流通网络工程”稳步推进，新建连锁农家店1648个、村级综合服务社748家。2011年社会消费品零售总额比2006年增长1.5倍。金融业健康发展，兴业、浦发、汉口等9家银行相继入驻宜昌。金融机构存贷款余额分别达到1751亿元和1213亿元，比2006年增长1.5倍和1.2倍。

　　这五年，是宜昌发展后劲显著增强的五年。着力转变发展方式，加大节能减排力度，单位生产总值能耗比2006年下降22%，化学需氧量和二氧化硫排放分别下降8%和4.8%，可持续发展能力不断增强。实行最严格的耕地保护制度，土地集约节约利用水平提高。大力实施沿江突破战略，统筹沿江开发区、园区建设与发展，沿江区域规模以上工业增加值超过全市的80%，成为全市经济发展的主要增长极。宜昌高新区升格为国家级高新区，形成“一区多园”构架。生物产业园、白洋工业园开发正式启动，高新区开发空间大为拓展。积极推进异地开发，兴发工业园等飞地园区健康发展，飞地经济成为山区、库区县经济发展的重要平台。加大项目建设力度，招商引资成果丰硕。三峡全通涂镀板、东阳光工业园、中兴汽车、联邦电缆、万达广场等一批投资过100亿、过10亿的重大项目落户宜昌。五年完成全社会固定资产投资3760亿元，是上一个五年的2.7倍。兴建了一批打基础、利长远的产业项目和基础设施项目，开工建设投资过5000万元的项目692个，有576个项目建成投产，为全市经济社会持续发展提供了后劲支撑。

　　这五年，是宜昌改革开放深入推进的五年。坚持深化改革，扩大开放，推进体制机制创新，经济社会发展活力进一步释放。国有企业改革不断深化，现代企业制度更趋完善，实现了国有资本保值增值。多种所有制经济竞相发展，民营经济成为市域经济最活跃的因素。创新投融资体制，投资公司、担保公司等融资平台实力增强。宜昌交运成功上市。市城投公司发行债券10亿元。已上市企业增发融资40.7亿元。农村公益性服务“以钱养事”新机制基本建立。农村集体林权制度、土地流转制度、农村金融改革逐步深化。医药卫生体制改革初见成效。新一轮政府机构改革全面完成。现代公共财政体系不断完善。社会管理创新试点经验在全国推广。人口和计划生育综合改革成为全国示范。对内对外开放成绩斐然。五年引进市外投资1050亿元，引进专家、博士等高层次人才650人。三峡机场航空口岸开通运行。2011年实现出口17亿美元，比2006年增长3.2倍。三峡库区对口支援工作卓有成效，五年落实援助资金294.3亿元。

　　这五年，是宜昌城乡面貌巨大变化的五年。坚持用市场办法推进城市扩容提质，城市综合服务能力增强。立体交通网络基本形成，区域性交通中心地位逐步显现。沪渝、荆宜、江南三峡翻坝高速公路和宜万铁路建成通车。宜巴、保宜高速公路和汉宜高速铁路正在加紧建设。全市高速公路总里程达到392公里，铁路总里程440公里，港口年吞吐量4600万吨，85%的县市区通达高速或一级公路，95%的县乡道路达到四级以上标准，所有行政村通达沥青或水泥路。城市规模不断扩大，功能日臻完善。五年筹资95亿元，开工建设城市基础设施项目200个。新建和改造中心城区道路245公里、城市排水排污管道420公里，沿江大道延伸段、城东大道、发展大道、临江溪污水处理厂等一批项目相继建成。城区垃圾无害化处理率达到91%，污水处理率90%。生态建设加强，全市森林覆盖率58%，城区集中式饮用水水源地水质达标率100%。城区新增园林绿地1013公顷，人均公园绿地面积11平方米。城镇化进程加快，城镇化率提高到50.8%。城区建成区面积拓展到110平方公里，常住人口超过100万。宜都、秭归、兴山获得“国家园林城”称号。秭归县城、夷陵军田坝村、宜都鸡头山村进入“全国文明村镇”行列。五峰县城回迁正式启动。聂家河、潘家湾、樟村坪被评为“全国环境优美乡镇”。新农村建设扎实推进，55个村被纳入全省新农村建设示范村。新增“一池三改”农户21.9万户，农村人居环境普遍改善。

　　这五年，是宜昌社会事业全面进步的五年。大力实施科教兴市战略，教育事业得到优先发展。九年义务教育完成率达到99%。高中阶段教育基本普及。职业教育加快发展，宜昌职教园开工建设。高等教育实力增强，三峡大学成为水电特色鲜明的综合性大学。文化事业取得长足进步，有5个县市区被评为全国文化先进县市区。文化基础设施日趋完善，宜昌广电中心、新宜昌图书馆、新宜昌剧院建成使用，改造、新建乡镇文化站91个，建成农家书屋1451个。广播电视实现村村通，综合覆盖率达到98%。文艺创作成绩显著，《楚水巴山》等多件作品获国家级文化奖。群众文化活动丰富多彩，荣获“全国集邮文化先进城市”称号，8个县乡被评为“中国民间文化艺术之乡”。新闻传媒、演艺娱乐、图书音像等文化产业方兴未艾，2011年实现增加值60亿元。医疗卫生服务体系建设加强，改造提升市县级医疗机构61家、乡镇卫生院99家，城镇社区全部配建卫生服务机构，99%的村卫生室达到甲级标准。新型农村合作医疗、国家基本药物制度实现全覆盖。5.8万残疾人接受康复服务，成为全国残疾人工作示范城市。群众健身运动蓬勃开展，竞技体育水平不断提高。对台、侨务、外事、宗教、保密、信访、档案、方志、妇女、儿童等各项工作都取得新成绩，气象、防震减灾、人防、老龄等各项事业都取得新进步。同时，民族工作、援藏援疆工作深入开展；国防动员、民兵预备役建设和“双拥”工作不断加强，连续四届荣获“全国双拥模范城”称号，军政军民关系更加密切。

　　这五年，是宜昌政务环境不断优化的五年。坚持依法行政，加强效能建设，着力提高政府执行力和公信力，形成了风清气正、团结实干的浓厚氛围。不断完善科学民主决策机制，加快推行电子政务，行政权力运行更加高效透明。行政审批提速提效，审批环节减少20%，审批时限压缩68%。行政监察和审计监督得到加强，党风廉政建设责任制深入落实。市政府及其部门认真听取人大代表、政协委员的意见和建议，五年按期办复人大代表建议1454件、政协委员提案1995件，办结率100%。“五五”普法效果良好，市民法律意识普遍提高。社会治安综合治理力度加大，连续两届被评为“全国社会治安综合治理优秀市”。突发公共事件应急处置能力增强。安全生产形势总体稳定。社会更加安定和谐。

　　这五年，是宜昌人民生活明显改善的五年。劳动保障和就业工作加强。新增城镇就业46万人，新增农村劳动力转移就业44万人，城镇登记失业率控制在4.3%以内。五项保险参保人数扩大到351.2万人次。有效解决了被征地农民、超龄职工等群体养老保险以及“关改破”困难企业职工医疗保险、农民工工伤保险等问题。城镇职工医保政策不断完善，城镇居民医保待遇大幅提高。城乡居民社会养老保险试点有序推进，参保人数达到108万人。落实社会救助资金20.4亿元。城镇低保应保尽保，农村低保扩大到16万人。城镇住房保障体系逐步健全，低收入群体住房困难得到缓解。建设经济适用房8249套、廉租房14640套，改造棚户区19027户，发放廉租房租赁补贴6623万元。城镇人均住房面积达到33平方米。落实各项惠农补贴30.3亿元。改造农村危房1.8万户。解决了103万人饮水安全问题。减少农村低收入和贫困人口13.3万人。落实民族地区对口帮扶资金7000万元。城乡居民家庭财富普遍增加，手机、电脑逐步普及，小汽车进入普通家庭。人均预期寿命达到74.5岁。

　　各位代表，过去的五年，宜昌发展成就斐然。我们成功应对国际金融危机严峻挑战，创造了“宜昌速度”，实现了弯道超越。我们举全市之力服务三峡工程，移民搬迁安置顺利完成，有力支持和保障了三峡工程全面蓄水发电。我们深入推进“三城联创”，在全省率先获得“国家卫生城市”、“国家环保模范城市”和“全国文明城市”殊荣，城市文明程度极大提高。这一切成绩的取得，都归功于省委省政府和市委正确领导，归功于全市人民共同努力，得益于历届市委市政府打下的良好基础，与社会各界大力支持、积极参与密不可分。在此，我代表市人民政府，向全市广大干部群众，向给予政府工作大力支持和有效监督的人大代表、政协委员和各民主党派、工商联、无党派人士，向在宜中省单位和广大外来投资者，向驻宜解放军、武警官兵，向所有关心支持宜昌建设和发展的同志们、朋友们，表示衷心感谢，并致以崇高敬意！

　　各位代表，五年的成绩来之不易，积累的经验更是难能可贵。我们深切体会到，要把宜昌改革、发展和建设事业推向前进，必须坚持科学发展，始终把产业培育和项目建设摆在经济工作首位；必须坚持解放思想，用改革创新的办法解决前进中的困难和问题；必须坚持服务优先，用良好环境凝聚发展合力；必须坚持依靠群众，靠团结实干成就事业；必须坚持以人为本，让发展成果惠及全体人民。

　　各位代表，我们认识到，发展不够仍然是宜昌最大的实际，全市综合实力离省域副中心城市要求还有较大差距。国际金融危机影响深远，外部环境更具复杂性和不确定性。全市经济增长的资源环境约束进一步显现，经济结构优化升级任务艰巨；制约科学发展的体制机制障碍仍然存在；政府服务、治理能力和机关办事效率有待进一步提高。对这些困难和问题，我们将勇于面对，不断改进和创新工作方法，采取有效措施，切实加以解决。

　　二、今后五年奋斗目标和主要任务

　　宜昌正处在发展黄金机遇期、城市快速扩张期和经济加速期，加快发展的有利因素很多。国家大力实施中部崛起战略，加大三峡库区后续发展扶持力度，加大武陵山区域发展和扶贫攻坚力度，推进东部沿海产业向内地转移。省委省政府全面实施“两圈一带”、“一主两副”战略，全力支持省域副中心城市建设。这些政策机遇正在宜昌产生叠加效应。宜昌区域性交通枢纽地位基本形成，为加快发展提供了重要支撑。近年来，一批重大项目陆续落户宜昌，蓄势待发，增添了发展后劲。全市上下政通人和，干部群众在实践中创造了很多好思路、好经验，成为宝贵的精神财富。尤其可喜的是，刚刚闭幕的市第五次党代会，科学描绘了今后五年宏伟蓝图，鼓舞人心，催人奋进，必将激发全市人民推动科学发展、跨越发展的强大活力与动力。

　　站在新的历史起点，我们对宜昌的发展充满信心。市政府将认真贯彻市第五次党代会精神，决不畏难，永不懈怠，克难奋进，乘势而上，以更加宽阔的视野，更加开放的思维，谋划宜昌未来，致力推动全市经济转型提速，社会事业全面进步。

　　今后五年，市政府工作的指导思想是：以邓小平理论和“三个代表”重要思想为指导，认真践行科学发展观，以科学发展为主题，以转变经济发展方式为主线，以改革开放创新为动力，以改善民生为目的，牢牢把握开放引领、沿江突破、创新驱动、绿色繁荣战略要点，加快构建现代产业体系，加快新型城镇化步伐，全面推进经济总量、城市发展、发展方式、社会建设新跨越，把宜昌建设成为省域副中心城市、长江中上游区域性中心城市和世界水电旅游名城，加快向现代化特大城市迈进。

　　奋斗目标是：到2016年，全市生产总值占全省比重达到15%左右，地方一般预算收入突破300亿元，全社会固定资产投资年均增长23%，社会消费品零售总额年均增长18%，城镇居民人均可支配收入和农民人均纯收入年均增长11%以上。

　　今后五年，市政府工作思路和措施主要是五个方面。

　　(一)产业第一，转型发展，增强城市支撑力

　　把握宜昌进入工业化中后期的阶段性特征和规律，以先进制造业为重点，推动工业做大做强；以旅游业和生产性服务业为重点，推动服务业转型升级，促进先进制造业和现代服务业双轮驱动，加快构建具有宜昌特色和比较优势的现代产业体系。力争工业增加值年均增长18%；第三产业增加值年均增长13%。

　　打造沿江万亿经济走廊。继续实施沿江突破战略，引导产业、项目、生产要素向沿江集中，集群集约发展，加快把沿江地区建设成年产值过万亿元的产业聚集区。充分发挥高新区龙头带动作用，统筹推进沿江开发，加快培育一批在全省乃至全国有影响力的特色专业化园区、一批具有较强竞争力的优势产业。力争2016年，高新区深圳工业园、白洋工业园等园区产值过1000亿；宜都、夷陵、当阳、枝江开发区产值过1000亿；电工电缆、汽车等一批特色园区产值过100亿；装备制造、化工、食品医药等支柱产业产值过1000亿。突破性发展新能源、新材料等战略性新兴产业，抢占发展制高点。深入实施中小企业成长工程和优强企业倍增工程，力争2016年，规模以上工业企业达到1500家，产值过100亿企业达到20家以上、过1000亿企业实现零的突破。抓好企业上市工作，力争新增上市企业5家以上。促进工业化与信息化融合，继续实施精品名牌战略，让更多“宜昌制造”唱响全国、走向世界。支持夷陵、宜都、枝江、当阳等东部市区率先发展，争取宜都、枝江早日进入全国县域经济百强。

　　推动服务业大发展大提升。适应新型工业化需要，突破性发展物流、金融、信息服务、服务外包等生产性服务业，壮大规模，完善体系，加快形成新的支柱产业。支持大型物流园区建设，推进多式联运，培育专业化物流企业，发展第三方物流，努力把宜昌建成三峡现代物流中心城市。支持各类金融机构发展壮大，推进农村金融全覆盖，争取更多金融资本投入宜昌，建设区域性金融中心。以“两坝一峡”区域为核心，以主城区为依托，整合旅游资源，倾力打造宜昌三峡国家级旅游度假区，做强三峡旅游核心品牌。推动清江画廊、柴埠溪等景区创建5A景区。高标准建设三峡游轮中心等一批重大旅游项目，完善旅游服务功能，推进旅游业转型升级，把宜昌建设成为国际旅游目的地城市。

　　大力发展现代农业。按照“特色、优质、高效”原则，深入推进农业结构战略性调整，优化农业区域布局，强化农业科技支撑，推进标准化生产，提升特色农产品市场竞争力。以工业化理念谋划农业，稳定粮食生产，着力打造柑橘、茶叶、蔬菜、畜牧、水产、食用油6个百亿产业，建成全国一流的柑橘主产区和茶叶强市。推进农产品加工向园区集中、向精深加工转型，不断增强农产品加工转化增值能力。力争2016年，农产品加工产值达到1200亿。精心培育农产品驰名商标、地理标志，做优农产品品牌。

　　(二)东拓南展，扩容提质，增强城市承载力

　　积极呼应武汉、重庆两大城市圈发展，实施沿江组团开发，优化城市空间布局，全面推进城市规模扩张、功能优化、品位提升，不断提高城市承载能力和综合服务能力，建设全国一流明星城市。

　　加快构建现代化特大城市基本框架。突出滨江山水园林特色，统筹城市与产业发展，推进中心城区东拓南展北联，规模扩张，力争2016年城区框架超过200平方公里。突破行政区划，实行共建共享，加快把白洋、火车东站片区建成产业新区、宜居新城。坚持规划引领、市场运作、分期开发，加快把点军、平湖半岛等区域建成生态城市新区。优化提升三峡坝区等外围组团。

　　加快完善城市综合服务功能。抓住国家发展内河水运机遇，实施以港兴城战略，整合岸线资源，提升集疏运能力，把宜昌组合港建设成年吞吐量过亿吨的三峡枢纽港。建成宜巴、保宜、紫云等一批高速公路和铁路，建成庙嘴、香溪、白洋等长江大桥，扩建三峡机场，不断完善水公铁空快速转换、无缝对接的大交通格局。完善城区路网结构，优先发展公共交通，确保城市畅通有序。加强给排水、电力电信、燃气管道等基础设施建设，确保配套功能适应城市发展步伐。推动电信网、广电网、互联网三网融合，加快城市信息化进程，促进城市现代化。

　　加快新型城镇化步伐。科学编制全地域规划，坚持城乡统筹，促进城乡基础设施共建共享、产业发展互动互促，实现中心城区、县城、小城镇、中心村协调发展。引导人口向城镇聚集，确保城镇化率达到56%以上。推进宜都、枝江、当阳向中等城市迈进，逐步与中心城区实现一体化发展。大力支持五峰县城回迁。依托长江、清江、香溪河、沮漳河等流域，推动城镇沿江沿河组团发展，形成滨水生态城镇带。加快新农村建设步伐，建成一批特色鲜明、文明富裕的中心村、示范村。

　　(三)创新驱动，开放引领，增强城市内生力

　　坚持不懈深化改革，扩大开放，加快制度、政策、科技创新步伐，形成更具活力、更加开放的经济体系，努力建设创新型城市。

　　不断创新有利于跨越发展的体制机制。营造全民创业、“草根经济”蓬勃发展的气候和土壤，支持非公有制经济加快发展。创新城市管理体制，合理划分市区两级财权与事权，逐步建立与现代化特大城市相适应的管理体制和运行机制。深化各项改革，充分发挥市场配置资源的基础性作用，让一切创造财富的源泉充分涌流，让一切推动发展的活力竞相迸发。

　　坚定不移扩大开放。坚持对内对外开放并举，放宽开放领域，提升开放层次，把宜昌建设成技术、资金、人才等要素聚集地。坚持人才强市战略，广泛吸引各类人才到宜昌发展。转变外贸发展方式，优化出口商品结构，扩大出口规模，提高出口产品竞争优势和效益。

　　切实增强自主创新能力。整合资源，建立健全以企业为主体、以市场为导向、政产学研相结合的创新体系。支持骨干企业特别是高新技术企业加强工程技术中心、企业技术中心、重点实验室等创新平台建设。实施知识产权战略，走差异化研发道路，支持研究机构围绕宜昌特色优势产业，加强核心技术、自主产品研发，促进科技成果转化，提升产品核心竞争力。

　　(四)繁荣文化，优化环境，增强城市软实力

　　抓住国家推动文化大发展大繁荣机遇，巩固提升全国文明城市创建成果，塑造城市文化灵魂，创新管理，优化环境，不断提升城市知名度、美誉度。

　　着力打造宜昌文化品牌。加快文化体制改革创新，推进文化事业和文化产业协调发展，不断满足人民群众日益增长的精神文化需求。新建宜昌博物馆、市文化活动中心等一批重点设施，增强公共文化服务功能。大力发展文化创意、影视传媒、数字动漫等文化产业，打造一批竞争力强的骨干企业和文化品牌。充分挖掘地域特色文化内涵，打造文艺精品，促进文化与旅游深度融合，增强宜昌特色文化生命力和影响力。

　　着力营造良好发展环境。锲而不舍地抓好软环境建设，切实形成公平公正的政策环境、规范严明的法制环境和务实高效的服务环境。大力弘扬“开放包容、务实创新、文明诚信”的城市精神，打造“宜人之城、昌盛之地”城市品牌。大力倡导重商、亲商、安商的文化氛围，让企业家备受欢迎、纳税人备受尊重，确保投资者进得来、留得住、发展好。

　　着力提升城市文明程度。不断推进社会管理创新，增进社区、邻里幸福和谐。维护社会稳定，建设“平安宜昌”。强化宣传引导，加强未成年人思想道德建设。深入开展群众性精神文明创建活动，着力提高市民文明素质，营造良好人文环境。建立长效机制，让“全国文明城市”、“国家卫生城市”和“国家环保模范城市”创建理念深入人心，让我们的城市更加安全有序、整洁美观、文明和谐、生态宜居。

　　(五)关注民生，成果共享，增强城市吸引力

　　把维护好、实现好、发展好人民群众的根本利益作为政府一切工作的出发点和落脚点，从人民群众最关心最直接最现实的问题入手，加大公共财政投入，切实改善民生，让广大市民分享改革发展成果。

　　始终把扩大就业摆在经济社会发展的优先位置。全面落实就业扶持政策，完善就业服务体系，最大限度地扩大城乡就业。健全困难群体就业援助制度，确保每个城镇家庭至少有1人就业。多层次开展职业技能培训。多渠道开发就业岗位。鼓励自主创业和自谋职业。建立稳定的工资增长机制，逐步提高最低工资标准，减少低收入群体，扩大中等收入群体，缩小贫富差距。确保5年新增城镇就业35万人，新增农村劳动力转移就业35万人。

　　协调发展各项社会事业。加大现代化教育强市建设力度。基本普及学前教育，促进义务教育县域均衡发展，高水平普及高中阶段教育。高标准建设宜昌职教园。支持三峡职业技术学院建成湖北一流、全国知名的高职院校。支持三峡大学建设水电特色鲜明的高水平综合性大学。深化医药卫生体制改革，建立覆盖城乡的基本医疗卫生制度，让群众享有安全、有效、方便、实惠的医疗卫生服务。坚持计划生育基本国策，促进人口均衡发展。推进农村广播电视“户户通”。加大群众体育设施建设投入，建设市奥林匹克中心，推进全民健身计划，提高市民健康水平。

　　着力完善城乡社会保障体系。全面落实惠及各类群体的社保政策，扩大社保覆盖面，提高保障水平。探索各类人群社会保险对接机制，逐步实现城镇医保和新农合可转换、一体化。建立城乡低保标准合理增长机制，逐步提高城镇低保标准，实现农村低保应保尽保。加强保障性住房建设，提高住房保障水平。完善专项扶贫、行业扶贫、社会扶贫三位一体的大扶贫格局，确保长阳、秭归、五峰农民人均纯收入增幅高于全国平均水平。积极应对人口老龄化，加强城乡养老服务机构建设，健全社会化养老服务体系。

　　各位代表，加快发展、改善民生是政府工作的不懈追求。市政府将和全市人民一道，经过五年奋斗，让宜昌的经济更加强盛，文化更加繁荣，社会更加和谐，生态更加美好，人民更加幸福。

　　三、2012年工作重点

　　2012年是实施“十二五”规划承上启下的重要一年，我们党将召开十八大，做好今年工作、实现新一届政府工作良好开局，对于推动经济社会跨越式发展，具有十分重要的意义。今年，全市经济社会发展主要预期是：生产总值增长13%，全社会固定资产投资增长23%，社会消费品零售总额增长18%，地方一般预算收入增长15%以上，外贸出口增长15%，城镇居民人均可支配收入和农民人均纯收入增长11%，城镇登记失业率控制在4.2%以内，全面完成省政府下达的节能减排任务，努力保持经济快速发展和物价水平基本稳定，保持社会和谐稳定，以优异成绩迎接党的十八大胜利召开。制定这些目标，充分考虑了发展基础和跨越要求，是积极进取并切实可行的。围绕这些目标，市政府将重点抓好七个方面的工作。

　　(一)坚持把项目建设摆在经济工作首位

　　统筹推进产业、基础设施和民生项目建设，确保投资快速增长，靠项目增量推动发展提速。

　　精心抓好项目策划与营销。组织推动各方力量，有效对接国家、省发展规划，着眼于产业培育和结构升级，精心策划一批后续产业项目和基础设施项目，充实项目库，确保储备项目投资总额突破2万亿元，确保今年新谋划投资过100亿元的项目10个以上。强化项目可研深度，缩短项目落地周期。对储备项目加强推介营销，提高项目知晓度和吸引力。

　　加大招商引资力度。推进市县两级招商联动，实行项目信息、客商信息资源共享。创新招商引资方式，大力推进园区招商、产业招商、以商招商，提高招商引资实效。瞄准珠三角、长三角、环渤海等发达地区，捕捉大企业、大集团投资意向，积极开展招商对接。围绕有利于改造传统产业、培植新兴产业、壮大支柱产业的大项目、好项目，着力引进战略投资者，力争在工业、城市基础设施、文化旅游、高端物流、金融商务、农产品深加工等领域实现招商新突破。加强与中省企业战略合作，支持磁电子产业园、中船重工江南工业园等加快建设。鼓励本土企业通过增资扩能、战略合作等途径，扩大规模，增强实力。力争宜昌高新区生物产业园落户项目20个以上，白洋和猇亭园区落户项目60个以上。积极争取三峡对口支援项目和经济协作项目，力争到位资金突破50亿元。确保全年招商引资实际到位资金450亿元以上。

　　扎实推进重点项目建设。完善项目工作机制，狠抓项目开工、投产。对重点项目继续实行领导联系制度，全程跟踪服务，及时解决好项目用地、能源、资金、用工等要素问题。明确各部门服务项目职责，畅通项目建设绿色通道，确保拟开工项目早日开工、在建项目早日建成、建成项目早日达产。重点支持河北中兴年产20万辆SUV系列整车、南玻年产700兆瓦硅片、华强科技非PVC多层共挤膜、力佳科技年产10亿只锂电池、东莞拓朴纳米新材料、安琪1万吨生物复合调味料等项目加快建设，支持联邦电缆高分子材料、人福药业出口药品生产基地等项目尽快开工。扎实做好白洋金帆达石化及新材料、中化化工园等项目前期工作。

　　(二)着力提高工业经济质量和效益

　　坚定不移实施工业强市战略，做大、做优、做强工业经济，夯实跨越发展的核心支撑。

　　加速推进优势产业规模扩张。科学制定千亿产业年度发展目标，细化推进措施，力争今年化工产业产值突破1000亿元、装备制造突破800亿元、食品医药突破750亿元。创新产业投入机制，推动科技、技改等专项资金投向产业培育的关键环节。扶持太阳能光伏、磁电子、纳米材料等战略性新兴产业加快发展，延伸产业链，尽快形成新的产业支柱。确保高新技术产业增加值增长30%。引导创新要素向优势企业聚集，做强自主创新平台，确保新增国家高新技术企业5家、省级以上企业工程技术中心2家。加大技术攻关力度，对关系产业发展的60个共性技术研发项目，实行公开招标，推进企业、产业技术进步。

　　加速推进企业做大做强。选择30家产业龙头企业，有针对性地实施优强企业倍增工程，力争新增主营业务收入过50亿元企业2家以上、过10亿元4家以上。全面落实国家各项扶持政策，研究制定配套措施，支持中小企业上规模上档次，促进小型微型企业快速成长。充分发挥经济工作部门、工商联、行业协会作用，积极搭建银企联手平台，切实缓解中小企业资金难题。强化“小进规”目标考核，建立健全奖惩办法，确保新增规模以上工业企业80家。完善激励机制，推进全民创业，鼓励民营业主“二次创业”。引导企业积极开发和采用先进技术，发展循环经济和低碳经济。大力实施质量兴市战略，支持企业争创精品名牌，确保新增中国驰名商标2件以上。加强煤、电、油、气、运等要素协调，保证企业正常生产经营。加强现代企业制度建设，引导企业抓好营销、财务、生产等内部管理，靠管理降成本、增效益、强实力。

　　加速推进开发区、园区开放开发。抓紧完善高新区各专业园区详规，以规划为龙头，推进园区集约节约开发。积极利用BT、BOT、发行债券等市场化融资方式，加快园区基础设施建设，改善项目落地条件。采取征转分离、先征后转，加大园区征地拆迁、土地开发力度，力争白洋工业园沙湾片区、田家河片区分别完成土地平整5平方公里，完成生物产业园土地平整5平方公里。开工建设白洋工业园田家河路、马家铺路、梅子溪路和园区污水处理厂，力争田家河港开工建设。确保生物产业园城乡路二期、花溪路、花艳一路、东站路、汉宜路改线段上半年竣工，东站路上段、东山三路、魏家畈变电站和污水处理厂等项目年内开工。大力支持科技企业孵化器发展。加快三峡云计算中心建设，争取纳入国家物联网云计算试点示范。加强出口基地建设，积极培育生物医药、装备制造等出口新优势。

　　(三)突破性发展现代服务业

　　把发展现代服务业作为结构调整的战略举措，重点发展旅游业和生产性服务业，推动服务业转型提速，确保第三产业增加值增长13%以上。

　　着力推进旅游业转型增效。把创建三峡国家级旅游度假区作为旅游业转型升级的突破口，尽快完成度假区总体规划编制，抓紧编制旅游码头、平湖半岛、三峡坝区和三峡人家修建性详规。完善“两坝一峡”区域监管体制。引导市场主体整合景区、游船、旅游码头等资源，构建发展共同体。加快平湖半岛旅游新区开发步伐。加快长江三峡游轮中心等重点项目建设。精心打造“两坝一峡”游标准化产品。不断完善城区文化娱乐、休闲度假功能，延长游客在宜停留时间。利用汉宜高速铁路开通机遇，加大旅游营销力度，鼓励旅行社组织来宜旅游专列、包机，拓展客源市场。支持清江画廊创建国家5Α景区。支持长阳、夷陵、五峰、秭归创建中国旅游强县。支持秭归归州镇、兴山昭君镇、远安荷花镇、夷陵石牌村等创建湖北旅游名镇名村。

　　加快三峡现代物流中心建设。围绕三峡现代物流中心发展规划，抓紧编制重点物流园区建设详规，研究制定鼓励制造业与物流业联动发展的相关政策，依托大交通，发展大物流。大力推进多式联运，畅通港口、公路、铁路、民用航空之间交通衔接。支持火车东站物流园、秭归三峡翻坝物流园等项目加快建设，建成一批布局合理、用地节约、产业集聚、功能集成、经营集约的物流园区。积极引进国内外优强物流企业，发展第三方物流，不断延伸仓储、加工、包装、配送、批发等物流产业链。建立健全物流公共信息平台，引导更多企业加入物流信息网。支持金东山市场、华祥商业中心、国贸集团等商贸流通企业创新业态，加快发展，增强辐射能力。

　　加快发展文化、金融等服务业。进一步深化文化体制改革。发展壮大出版、印刷、广告、演艺、娱乐等传统文化产业，加快发展文化创意、数字动漫等新型文化产业。鼓励有实力的文化企业跨地区、跨行业、跨所有制兼并重组，做大做强。抓紧启动宜昌文化创意产业园建设。支持三峡日报传媒集团等文化企业拓展市场空间，延伸业务领域。着力引进各类金融机构到宜昌设立区域性总部。鼓励有条件的金融机构创办村镇银行。完善激励政策，积极引进培育风险投资机构。支持有实力的企业组建创业投资、风险投资、小额贷款公司等金融实体。发挥财政资金“四两拨千斤”作用，充实担保公司资本金，壮大融资能力。加快组建三峡农村商业银行。支持东阳光药业、匡通电子等企业加快上市步伐。支持市财政经济开发投资公司发行企业债券，支持市城投公司再发融资债券。实施中小商贸企业成长工程，力争新增限额以上商贸企业300家。大力发展信息服务、科技服务、商务服务，打造服务外包载体，推进服务外包产业发展。积极发展体育健身、教育培训、家政服务等生活性服务业，拓展新的消费空间。继续实施“万村千乡市场工程”和“新农村现代流通网络工程”，新建连锁农家店120个、村级综合服务社200家，改善农村消费环境。

　　(四)千方百计促进农业增效农民增收

　　坚持走特色发展之路，进一步夯实农业基础，优化农业结构，推进农产品精深加工，实现特色农业提质增效，确保全年农业增加值增长4%以上。

　　深入推进特色农业产业化。强化农业科技推广，大力实施标准化生产，建设畜禽、水产、蔬菜标准化示范基地42个，新增标准果园10万亩、高效茶园5万亩、安全菜园3万亩、木本油料15万亩。实施新一轮城郊菜蓝子工程，新发展城郊蔬菜基地1万亩。探索农产品可追溯制度，确保农产品质量安全。促进产销对接、农超对接，畅通农产品销售渠道。加大政策、资金扶持力度，深入推进农产品加工“四个一批”工程，积极培育产业关联度高、带动力强的骨干龙头企业，力争新增规模以上农产品加工企业20家，9家企业产值过10亿元。规范发展农民专业合作社，提高农民组织化程度。引导龙头企业通过定向投入、委托生产、保护价收购、入股分红等多种形式，与农户结成利益共同体。

　　改善农村生产生活条件。把握国家政策机遇，统筹谋划并争取一批农田水利、土地整理、农业综合开发等重大项目。加紧启动沮漳河防洪治理工程。推进中小河流综合治理。完成东风渠等灌区节水改造和配套建设年度任务。加强“小农水”项目建设，新建、改造末级渠系500公里。扎实抓好“万名干部进万村挖万塘”活动，整治堰塘8500口。治理水土流失80平方公里。完成66座小型病险水库除险加固任务。解决10万人以上饮水安全问题。改造县乡公路230公里，新建通村油路500公里。加强生态家园建设，完成农村清洁能源入户工程3.5万户。改造农村危房1.5万户。推进集中连片特困地区扶贫攻坚，将年人均纯收入不足2300元的群众全部纳入扶贫范围，加大整村推进扶贫开发力度。根据国家《武陵山片区区域发展与扶贫攻坚规划》，积极争取项目，抓紧编制实施规划，加快五峰、长阳、秭归脱贫奔小康步伐。认真落实三峡后续工作规划，积极扶持库区移民生产发展，促进移民安稳致富。

　　着力拓宽农民增收渠道。全面落实强农惠农各项政策，增加农民政策性收入。整合培训资源，开展农民培训和农村实用人才开发，提高农民科技水平和转移就业能力。积极发展农村非农产业，促进农民就近转移就业，增加工资性收入。建立完善农村土地经营权、林权流转和抵押机制，增加农民财产性收入。

　　(五)继续加强城乡基础设施建设

　　坚持政府主导，市场运作，城乡统筹，建设一批交通、市政等基础设施，拓展城市框架。

　　加快完善综合交通功能。抓紧改建318国道、小鸦等一级公路。确保陆渔一级公路全线通车。开工建设当枝等一批一级公路。争取庙嘴、香溪长江大桥尽早开工。认真做好白洋长江大桥前期工作。支持宜巴、保宜高速公路加快建设。切实做好宜昌至张家界、太平溪至张家口高速公路前期工作。加快云池港二期、峡口等码头建设，确保白洋港一期、茅坪港二期年内开工。加紧做好枝城配煤中心、红花套、枝江等综合码头前期工作。启动三峡库区支流航道建设。加快整治黄柏河航道。加快建设紫云地方铁路。

　　加大城区基础设施建设力度。围绕城市总体规划，组织编制城市近期建设规划、城市新区建设规划和基础设施专项规划。力争开工建设葛洲坝至宜昌长江公路大桥江南一级公路，有序推进江南新区开发。加快城市新区给排水、供电、供气等管网建设。抓好东山四路、西陵二路延伸段等重点项目建设，推进运河、合益沟综合整治。确保宜昌汽车客运中心、东山高速客运站建成营运。开工扩建猇亭供水厂，确保猇亭污水处理厂年内竣工。继续实施城区夜景灯光工程。

　　着力改善人居环境。加强生态建设，确保实现国家森林城市创建目标。加大城区绿化力度，确保绿地率稳步增长。新建猇亭、点军垃圾填埋场。规范建筑垃圾管理。推进餐厨垃圾无害化处理。探索实施城区垃圾分类收集处理。加强清江、黄柏河、香溪河、柏临河、渔洋河等重点流域环境治理。加强城镇集中式饮用水水源地保护。按照国家颁布的城市空气质量新标准，探索推进大气污染联防联控，加强机动车尾气污染防治。基本完成城区背街小巷治理和菜市场标准化改造。加强城区交通节点、停车场建设和小区停车管理。加大城市环境综合治理力度，基本完成户外广告综合整治。全面推行数字化城市管理，确保城管执法全天候、全覆盖、无缝隙。

　　(六)切实抓好关系民生的各项工作

　　今年，市财政将进一步优化支出结构，加大民生投入，改进公共服务，切实把涉及民生的事项办实办好，让人民群众更加满意。

　　加快教育、卫生等社会事业发展。提高教育支出占财政支出的比重，优先发展各级各类教育。改善学前教育办学条件，加快义务教育现代化学校建设步伐。基本完成宜昌职教园建设。积极化解高校债务。加快区域性医疗卫生中心建设，支持重点医院争优创名，增强区域服务能力。合理布局医疗资源，完善社区、乡村医疗服务网络，满足群众基本医疗卫生需求。筹建新宜昌博物馆和文化艺术馆。实施文化信息资源共享、农家书屋等惠民工程，促进公共文化设施向基层倾斜。加快社会科学、广播电视、新闻出版等文化事业发展。激励文艺创作，力争推出更多优秀作品。

　　下大力拓宽就业渠道。全面落实就业创业扶持政策，新增小额担保贷款发放1.8亿元，大力支持自主创业。重点做好大学毕业生、返乡农民工、就业困难群众就业工作。继续开展“志能”激发关爱活动。建立健全“零就业家庭”就业援助长效机制。开展各类职业技术培训和技能竞赛，提高求职者和在岗职工职业技能水平，推进各类群体稳定、高质量就业。确保新增城镇就业7万人，下岗失业人员和困难人员再就业2.5万人，农村劳动力转移就业7.5万人。强化劳动监察与维权，确保劳动合同签订率达到90%。

　　加强社会保障体系建设。完善社保政策，降低社保门槛，扩大社会保险覆盖面，确保扩面18.3万人次。实行医疗、工伤、生育保险市级统筹。完善社会保险跨地区转移制度和不同人群社会保险衔接制度。全面推进城乡居民社会养老保险工作，实现社会养老保险制度全覆盖，确保参保续保率达到80%以上。完善新型农村合作医疗制度，确保参合率保持在95%以上、住院补偿比例超过70%。将新型农村合作医疗和城镇居民基本医疗保险人均补助标准提高到240元。扩大农村居民重大疾病医疗保障试点病种范围，全面推行农村儿童先天性心脏病、白血病大病保障。建设30个农村空巢老人日间照料中心。加快保障性安居工程建设，建设保障性住房33061套。认真贯彻国家《国有土地上房屋征收与补偿条例》，力争实施城区旧城改造77万平方米，改造危旧房8000余套。落实好廉租住房补贴的各项政策。筹集资金1.5亿元，帮助困难群众和优抚对象解决各种临时性生活困难。

　　切实维护社会和谐稳定。做好全国社会管理创新综合试点各项工作，完善网格化管理、信息化支撑、全程化服务体系。全面推进社会信用体系建设。强化社会管理综合治理，加强信访和人民调解、行政调解、司法调解工作，完善社会稳定风险评估和矛盾排查化解机制，提高全社会危机管理和抗风险能力。维护三峡库坝区社会稳定，服务三峡工程安全运行。创新食品药品安全监管体制机制，努力创建食品安全放心城市。加强基层民主政治建设，不断提升城乡居民自治水平。完善法律援助机制，切实维护弱势群体合法权益。引导企业构建和谐劳动关系。推进安全标准化建设，严格落实安全生产责任，继续开展安全专项整治和隐患排查治理行动，提高安全保障水平。

　　同时，扎实推进国防动员工作，大力支持驻宜解放军和武警部队建设，不断提高预备役部队、民兵和人民防空建设水平。继续深入开展“双拥”创建活动，巩固军政军民团结。扎实做好援藏援疆工作。依法管理宗教事务。积极发展人口和计划生育、慈善、残疾人、老龄、气象、消防、防震、体育等社会事业。加强统计、粮食、对台、外事、侨务、档案、方志、保密等各项工作。

　　(七)进一步加强政府自身建设

　　全面履行政府职能，提高为经济社会发展服务、为人民服务的能力和水平，进一步树立“为民、务实、清廉、高效”的政府形象。

　　坚持依法行政。把依法行政贯穿政府决策、执行、监督的全过程和政府管理的各个环节，严格按照法定权限和程序行使权力、履行职责。健全群众参与、专家咨询和集体决策相结合的行政决策机制，推进决策科学化、民主化、法治化。改进和创新执法方式，推行执法流程网上管理，促进行政执法部门信息交流和资源共享。依法接受人大法律监督和工作监督。主动接受政协和各民主党派、工商联民主监督。自觉接受群众监督和新闻监督。推进政务公开，提高政府工作透明度。

　　提高行政效能。进一步减少行政审批事项，规范审批程序，优化审批流程。运用电子政务平台，全面推行网上审批、网上办公和电子监察。加强学习型机关建设，着力提高公务员创新创造能力、应对复杂局面能力。大兴调查研究、求真务实之风，精简会议和文件，减少不必要的应酬，把精力用在抓改革、谋发展、保稳定、促和谐上，用在解决群众生产生活的紧迫问题上。把“依法依规、打破常规、特事特办、优质服务”理念转化为制度规范，内化为干部的行为习惯。健全目标管理体系，确保各项工作责任到位、考核到位。强化政务督查和效能监察，扎实开展“治庸问责”。

　　强化廉政建设。加强政府投资项目监管，确保每项公共工程成为优质工程、廉洁工程。强化审计监督，做到财政资金运用到哪里，审计就跟进到哪里。厉行勤俭节约，反对铺张浪费，把财政资金用在推动发展、改善民生的关键环节。加强惩治和预防腐败体系建设，严肃查处各类违法违纪案件，切实解决损害群众利益的突出问题。

　　各位代表，宜昌已步入跨越发展的关键时期，历史激励着我们，时代召唤着我们，人民期待着我们。让我们紧密团结在以胡锦涛同志为总书记的党中央周围，在中共宜昌市委坚强领导下，深入落实科学发展观，勤勉精进，继往开来，为把宜昌建成省域副中心城市、长江中上游区域性中心城市和世界水电旅游名城而努力奋斗！
